# Is the Median Hourly Ambulatory Heart Rate Range Helpful in Stratifying Mortality Risk among Newly Diagnosed Atrial Fibrillation Patients?

**DOI:** 10.3390/jpm11111202

**Published:** 2021-11-14

**Authors:** Hsing-Yu Chen, John Malik, Hau-Tieng Wu, Chun-Li Wang

**Affiliations:** 1 Graduate Institute of Clinical Medical Sciences, College of Medicine, Chang Gung University, Taoyuan 33302, Taiwan; b8705016@gmail.com; 2 Division of Chinese Internal Medicine, Center for Traditional Chinese Medicine, Chang Gung Memorial Hospital, Taoyuan 33378, Taiwan; 3 School of Traditional Chinese Medicine, College of Medicine, Chang Gung University, Taoyuan 33302, Taiwan; 4 Department of Mathematics and Department of Statistical Science, Duke University, Durham, NC 27708, USA; malik.john@hotmail.com (J.M.); hauwu@math.duke.edu (H.-T.W.); 5 Mathematics Division, National Center for Theoretical Sciences, Taipei 106, Taiwan; 6 Linkou Medical Center, Cardiovascular Division, Department of Internal Medicine, Chang Gung Memorial Hospital, Taoyuan 33305, Taiwan; 7 College of Medicine, Chang Gung University, Taoyuan 33302, Taiwan

**Keywords:** all-cause mortality, atrial fibrillation, Holter monitor, the median hourly ambulatory heart rate range

## Abstract

**Background:** The application of heart rate variability is problematic in patients with atrial fibrillation (AF). This study aims to explore the associations between all-cause mortality and the median hourly ambulatory heart rate range (${\tilde{AHRR}}_{24hr}$) compared with other parameters obtained from the Holter monitor in patients with newly diagnosed AF. **Material and Methods:** A total of 30 parameters obtained from 521 persistent AF patients’ Holter monitor were analyzed retrospectively from 1 January 2010 to 31 July 2014. Every patient was followed up to the occurrence of death or the end of 30 June 2017. **Results:** ${\tilde{AHRR}}_{24hr}$ was the most feasible Holter parameter. Lower ${\tilde{AHRR}}_{24hr}$ was associated with increased risk of all-cause mortality (adjusted hazard ratio [aHR] for every 10-bpm reduction: 2.70, 95% confidence interval [CI]: 1.75–4.17, *p* < 0.001). The C-statistic of ${\tilde{AHRR}}_{24hr}$ alone was 0.707 (95% CI: 0.658–0.756), and 0.697 (95% CI: 0.650–0.744) for the CHA2DS2-VASc score alone. By combining ${\tilde{AHRR}}_{24hr}$ with the CHA2DS2-VASc score, the C-statistic could improve to 0.764 (95% CI: 0.722–0.806). While using 20 bpm as the cut-off value, the aHR was 3.66 (95% CI: 2.05–6.52) for patients with ${\tilde{AHRR}}_{24hr}$ < 20 bpm in contrast to patients with ${\tilde{AHRR}}_{24hr}$ ≥ 20 bpm. **Conclusions:** ${\tilde{AHRR}}_{24hr}$ could be helpful for risk stratification for AF in addition to the CHA2DS2-VASc score.

## 1. Introduction

Atrial fibrillation (AF), a common and severe rhythm disturbance, is associated with increased mortality and morbidity [1]. Several models based on clinical or monitor parameters have been used to predict the outcome of AF in different clinical settings [2,3,4,5,6]. The CHA2DS2-VASc score, including the existence of congestive heart failure (CHF), hypertension, aging, diabetes mellitus (DM), prior stroke or thromboembolism events, vascular diseases, and sex category, has been widely used to stratify the risk of stroke in patients with AF [2,7]. Recently, the association between the CHA2DS2-VASc score and the death of CHF patients with AF was also reported [8]. However, the utilization of AF instrumental monitors on the outcome prediction of AF is lacking. Heart rate variability (HRV) enables quantification of cardiac regulatory influences on the autonomic function and predicts the prognosis in patients with CHF, myocardial infarction (MI), and AF [6,9,10,11,12]. HRV can be improved following intervention and is associated with better outcomes [13,14]. Nonetheless, most HRV studies exclude patients who are not in sinus rhythm, and there have been relatively few published reports dealing with HRV in patients with AF^6^. Furthermore, the application of HRV is problematic in patients with AF due to the absence of sinoatrial node activities and the complexity of ventricular response in such patients. A few studies applied measurements of ventricular irregularity for predicting mortality in patients with AF, such as entropy measured from 24-h ambulatory ECG recordings, the heart rate variability triangular index (HRVI), and the square root of the mean squared difference of continuous normal-to-normal intervals (RMSSD) [6,15,16]. However, the complexity of analysis could be a significant deterrent for clinical application, and we hypothesized that there might be other simplified parameters to reflect the significance of HRV among AF patients. 

Recently, some simplified parameters obtained from the 24-h Holter electrocardiogram were introduced. Several epidemiological studies have shown that a higher resting heart rate is associated with an increased risk of death from cardiovascular and non-cardiovascular causes in middle-aged and older subjects [17,18]. Wang et al. showed that nighttime heart rate had more prognostic value than the resting HR and 24-h HR for all-cause mortality among patients with heart failure and reduced ejection fraction [19]. Moreover, the 24-h ambulatory heart rate range (24-h AHRR), proposed by Cubbon et al., predicts the risks of hospitalization and all-cause mortality among patients with HF, while about one-third of these patients had AF [20]. For AF, in addition to the abovementioned entropy-related parameters proposed by Yamada et al. [15], lower 24-h total heartbeats were associated with a higher incidence of cardiac death and heart failure hospitalization among permanent AF patients [21]. The hourly analysis may reflect quite different aspects of patients’ conditions, so we proposed that ${\tilde{AHRR}}_{24hr}$ may be more indicative among AF patients since the heart rate may fluctuate during several short periods [22]. HRV-related parameters were also examined among CHF patients with AF and myocardial infarction patients [12,16]. The feasibility of using HRV among patients with incident AF has not been tested, and the comparisons between HRV parameters and clinical scores are less reported. Additionally, the feasibility of using AHRR-related parameters and other parameters obtained from the Holter monitor for predicting the prognosis of AF remains unknown. 

This study aims to systematically discover the associations between all-cause mortality and the commonly used Holter and HRV parameters acquired from analysis on ventricular and atrial waveforms, such as 24-h AHRR, ${\tilde{AHRR}}_{24hr}$, coefficient of variation, concordance, sample entropy, singular values of the Poincaré plot, teager energy, RMSSD, the standard deviation of N-N intervals index (SDNNI), and the percentage of successive R-R intervals (pRR50) among the incident AF patients. Additionally, the comparisons of the prognostic value of AF patients between ${\tilde{AHRR}}_{24hr}$ and CHA2DS2-VASc were assessed to determine the possible role of using ${\tilde{AHRR}}_{24hr}$ among AF patients.

## 2. Material and Methods

### 2.1. Study Design and Subject Enrollment

**Figure 1:**
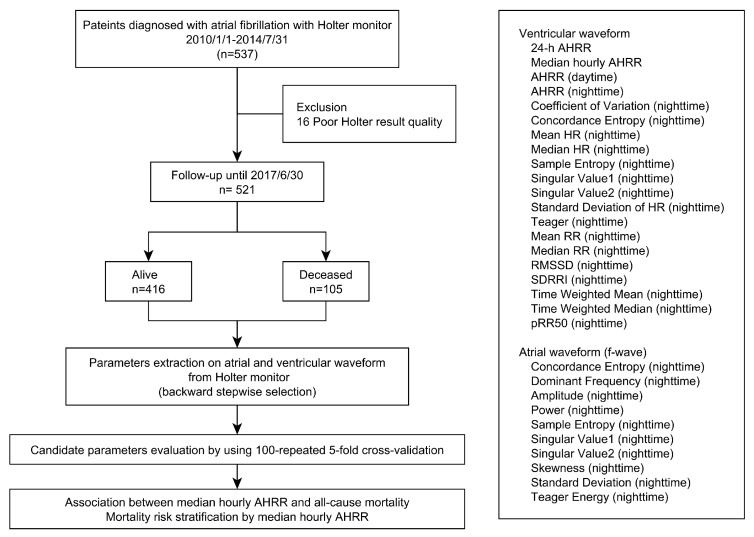
Flow diagram of this study.

Figure 1 demonstrates the study flowchart. We retrospectively collected 24-h Holter ECG recordings of 537 newly diagnosed persistent AF patients from the Chang Gung Memorial Hospital, Linkou Branch, Taiwan, between 1 January 2010 and 31 July 2014. The Philips Zymed Holter analysis software (Zymed DigiTrak Plus; Zymed 1810, Philips, Amsterdam, The Netherlands) was used for analyzing the 24-h ECG waveforms of each patient. If patients had more than one 24-h Holter recording, only the first ECG readings were analyzed. This study was conducted in a two-staged design: the first stage was to select the most potential parameters among various calculations from the analysis on the ventricular and atrial waveforms from the Holter monitor by assessing the associations between all-cause mortality and each parameter; and the second stage was to observe the feasibility of examining the relationships between the candidate parameter and all-cause mortality after considering clinical covariates. The entire protocol was approved by the institutional review board of the Chang Gung Medical Foundation (201800943B0C601). 

### 2.2. Holter Parameters Calculation

Based on the prior algorithm to process AF Holter monitor signals, estimations of Holter monitor parameters were obtained [23]. To test the quality of a given recording, we calculated the Fourier transform of the signal per minute to measure the presence of overwhelming noise. Any minute where the noise was found to be above a certain threshold was treated as missing data (*n* = 16, Figure 1). The ventricular activity in the 24-h recording is then recorded every minute, and the number of ventricular peaks occurring was counted. This number served to roughly indicate the heart rate over short periods. This method was chosen because of its apparent stability in the presence of imperfect R-peak detections. A series of 1440 heart-rate measurements for each subject were recorded and analyzed.

Various Holter parameters were extracted from the 24-h readings, and the calculation of the ${\tilde{AHRR}}_{24hr}$ is explained as follows as an example. The details of all Holter parameters are explained in Supplementary Materials File S1. 

Suppose there are N detected heartbeats in the ECG signal and that the location of the i-th heartbeat is $t_{i}$ (in seconds). A new time series, $H\in {\mathbb{R}}^{1440}$, is built so that

$$H\left(n\right)=\left|\left\{1\leq i\leq N:\left(n-1\right)60<t_{i}\leq n60\right\}\right|$$

for all 1≤n≤1440. That is, the n-th entry of H is the number of heartbeats that occurs during the n-th minute of the recording. The index AHRR (the traditional ambulatory heart rate range, adjusted for subjects with atrial fibrillation) is calculated as

$$\mathrm{AHRR}=\underset{1\leq n\leq 1440}{\max}H\left(n\right)-\underset{1\leq n\leq 1440}{\min}H\left(n\right)$$

The index of ${\tilde{AHRR}}_{24hr}$ is calculated by building a new time series $G\in {\mathbb{R}}^{24}$ so that

(1) G(k)=max{H(n):(k−1)60<n≤k60}−min{H(n):(k−1)60<n≤k60}

and the setting is

(2) $${\tilde{AHRR}}_{24hr}=\mathrm{Median}\left\{G\left(k\right):1\leq k\leq 24\right\}$$

The intention is that we calculate the median of a sequence of AHRR values that are estimated hourly. 

### 2.3. Demographic Covariates and Outcome

In addition to Holter parameters, demographic features around the date of the Holter ECG recording were collected, including age, gender, co-morbidities, medications, and laboratory findings. The CHA2DS2-VASc score was also calculated as an individual covariate due to its high associations with mortality among AF patients [2,24,25,26]. All-cause mortality was set as the primary endpoint, and every patient was followed until that point or 30 June 2017. Cardiovascular mortality, analyzed in the sensitivity test as a secondary outcome, was defined as death related to stroke, HF, ventricular arrhythmia, or pulmonary embolism.

### 2.4. Statistical Analysis

Descriptive statistics were used for demographic features and all Holter parameters, and student’s *t*-tests or χ^2^ tests were used to examine the differences between the living and deceased patients. We used a two-stage data processing flow to obtain the most potential Holter parameters and examine the associations between candidate parameters and all-cause mortality. In the first stage of data processing, univariate, multivariate, and backward stepwise selection by Cox regression were applied to all accessible Holter ECG parameters to screen out the candidates of the Holter parameters. In the second stage of data processing, C-statistics and hazard ratios of the candidate parameter with demographic features were used to examine the incremental associations between all-cause mortalities and candidate parameters. Furthermore, the cut-off value of the candidate parameter would be determined, and the outcome of subjects stratified by the cut-off value was assessed as well [27]. The Kaplan-Meier estimation of 5-year all-cause mortality and Cox regression with demographic covariate adjustment were carried out in the subject stratification. For sensitivity tests, cardiovascular mortality as the endpoint instead of all-cause mortality of all subjects, 1:1 subsampling with association evaluation, and 5-fold cross-validation with 100 repeats as internal validation tests were carried out. Overlap weighting was used to balance the baseline differences between the alive and deceased patients throughout the entire study. All analyses in this study were conducted by using STATA (StataCorp. 2019. Stata Statistical Software: Release 16. College Station, TX, USA: StataCorp LLC.). The statistics with *p* < 0.05 were thought to be significant. 

## 3. Results

### 3.1. Baseline Demographic Features of Enrollees

**Figure 2:**
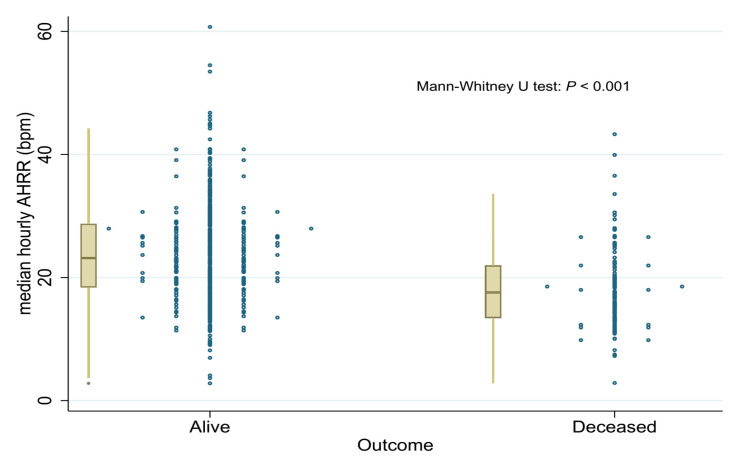
Distribution of median hourly AHRR (${\tilde{AHRR}}_{24hr}$).

Table 1 shows the baseline characteristics of the 521 studied patients. A total of 105 patients were deceased during the follow-up period (median: 47.7 months, interquartile range [IQR]: 31.6–61.9), accounting for 20.2% of all patients. Compared with the alive patients, the deceased were older (76.7 ± 11.4 years versus 67.6 ± 12.9 years, *p* < 0.001), had higher prevalence of heart failure (46.7% versus 25.3%, *p* < 0.001), stroke (27.6% versus 15.4%, *p* = 0.004), vascular diseases (20% versus 11.6%, *p* = 0.023), and higher CHA2DS2-VASc scores (4.2 ± 1.8 versus 2.8 ± 1.8, *p* < 0.001). For medication use, the deceased had lower prescription rates of anticoagulants (20% lower than the alive, *p* < 0.001) and β-blockers (15% lower, *p* = 0.002), but higher prevalence of using digoxin (11.5% higher, *p* = 0.033). Moreover, renal dysfunction was more commonly reported among the deceased, with their eGFR being about 20 mL/min lower (*p* < 0.001). Amongst 37 parameters calculated from the Holter monitor signals, four Holter parameters remained: ${\tilde{AHRR}}_{24hr}$, median, AHR at nighttime, singular value1 of f-waves at nighttime, and time-weighted median RR at nighttime (Supplementary Materials Files S2–S6). C-statistics were calculated for these four Holter parameters and two manually selected parameters: daytime and 24-h AHRR, which was reported previously and highly correlated to ${\tilde{AHRR}}_{24hr}$ (Supplementary Materials File S7). Finally, ${\tilde{AHRR}}_{24hr}$ was selected as the single Holter parameter candidate with the highest C-statistic (Supplementary Materials File S8). The ${\tilde{AHRR}}_{24hr}$ was significantly different among the alive and deceased patients (Supplementary Materials File S2), and it was significantly lower among the deceased (18.4 ± 6.8 bpm versus 24.0 ± 8.3 bpm, *p* < 0.001. Table 1 and Figure 2).

### 3.2. Associations of ${\tilde{AHRR}}_{24hr}$ and Baseline Demographic Features with All-Cause Mortality

**Figure 3:**
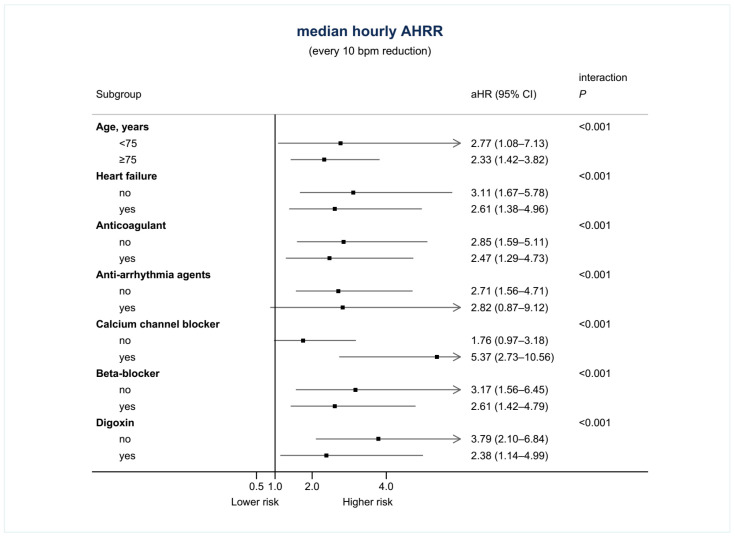
Stratified multivariate regression for median hourly AHRR (${\tilde{AHRR}}_{24hr}$).

**Figure 4:**
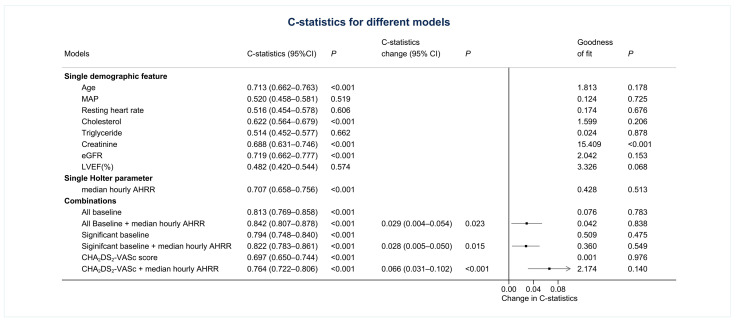
C-statistics of different models and the changes by applying median hourly AHRR (${\tilde{AHRR}}_{24hr}$) to demographic features. (Significant baseline parameters: Cholesterol, Digoxin, β-blockers, eGFR, anticoagulant, and CHA_2_DS_2_-VASc).

Under the consideration of all covariates, the use of only anticoagulants and digoxin significantly influenced the risk of mortality in the multivariate regression (adjusted hazard ratio [aHR]: 0.44 for the use of anticoagulants, 95% confidence interval [CI]: 0.28–0.69; aHR: 2.07 for the use of digoxin, 95% CI: 1.25–3.42). For ${\tilde{AHRR}}_{24hr}$, the difference was still significant when considering all demographic covariates, and the risk of all-cause mortality was 2.7-fold when ${\tilde{AHRR}}_{24hr}$ reduced by 10 bpm (95% CI: 1.75–4.17, *p* < 0.001) (Table 2). The trend was consistent across different age groups, groups with or without heart failure, use of anticoagulants, antiarrhythmic agents, calcium channel blockers, β-blockers, and digoxin (Figure 3). By using the C-statistic, ${\tilde{AHRR}}_{24hr}$ stood for an acceptable association with all-cause mortality as a single Holter parameter (0.707, 95% CI: 0.658–0.756, *p* < 0.001). Moreover, the use of ${\tilde{AHRR}}_{24hr}$ would positively increase the associations between all-cause mortality and models with demographic parameters, including all baseline demographic characteristics, significant demographic characteristics, and the CHA2DS2-VASc score alone (Figure 4). The improvement was most remarkable when combining ${\tilde{AHRR}}_{24hr}$ with the CHA2DS2-VASc score (0.066 higher than CHA2DS2-VASc score alone, 95% CI: 0.031–0.102, Figure 4). The advantages of using ${\tilde{AHRR}}_{24hr}$ were similarly found in sensitivity tests, including the associations with cardiovascular mortality, 1:1 subsampling before C-statistic evaluation, and 5-fold cross-validation with 100 repeats (Supplementary Materials File S8).

### 3.3. ${\tilde{AHRR}}_{24hr}$ Lower than 20 bpm was Associated with Higher All-Cause Mortality

**Figure 5:**
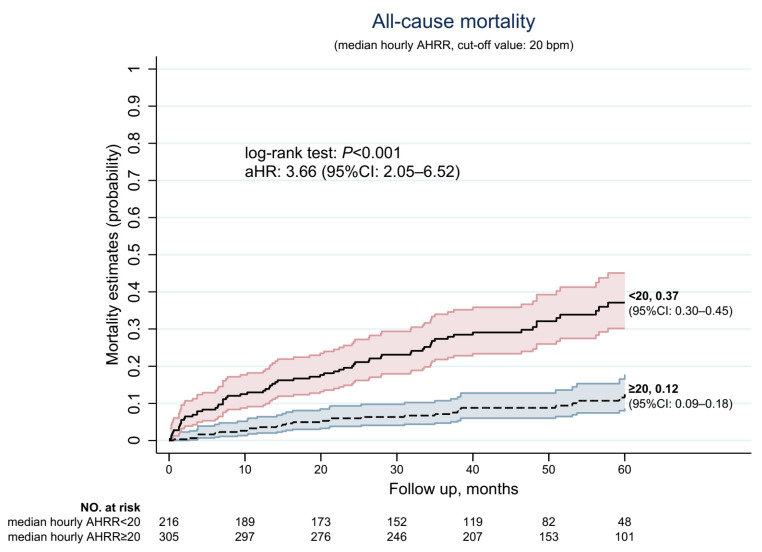
Accumulative incidence of 5-year all-cause mortality, stratified by median hourly AHRR (${\tilde{AHRR}}_{24hr}$) (cut-off value: 20 bpm).

The median value of ${\tilde{AHRR}}_{24hr}$ was 21.8 (IQR:17.0–27.8) and the optimal cut point was 19.1 (sensitivity: 0.68, specificity: 0.72). To test the feasibility of ${\tilde{AHRR}}_{24hr}$, the cut-off value was set to 20 bpm to stratify the risk of all-cause mortality among AF patients (sensitivity: 0.72, specificity: 0.65). Table 3 shows the differences between patients with ${\tilde{AHRR}}_{24hr}$. The outcome of these two groups of patients was quite different: patients with ${\tilde{AHRR}}_{24hr}$ < 20 bpm had a higher rate of all-cause and cardiovascular mortality (32.9% versus 11.1%, and 20.8% versus 6.6% of patients with ${\tilde{AHRR}}_{24hr}$ ≥ 20 bpm, *p* < 0.001). However, the medication use among these two groups was similar (*p* all > 0.05), although patients with ${\tilde{AHRR}}_{24hr}$ < 20 bpm had a higher prevalence of vascular diseases (9% higher than patients with ${\tilde{AHRR}}_{24hr}$ ≥ 20 bpm, *p* = 0.003) and hypertension (9.5% higher than patients with ${\tilde{AHRR}}_{24hr}$ ≥ 20 bpm, *p* = 0.028), and slightly higher CHA2DS2-VASc scores (0.7 points higher than patients with ${\tilde{AHRR}}_{24hr}$ ≥ 20 bpm, *p* < 0.001). Biochemical profiles were similar among these two groups, except the eGFR was 9.8 mL/min lower among patients with ${\tilde{AHRR}}_{24hr}$ < 20 bpm. The resting heart rate was 5.3 bpm lower than patients with ${\tilde{AHRR}}_{24hr}$ ≥ 20 bpm (*p* = 0.003). Figure 5 shows the survival estimation of these two groups of patients. For 5-year all-cause mortality, the probability of deceased status was 0.37 for patients with ${\tilde{AHRR}}_{24hr}$ < 20 bpm (95% CI: 0.30–0.45) and 0.12 for patients with ${\tilde{AHRR}}_{24hr}$ ≥ 20 bpm (95% CI: 0.09–0.18, log-rank *p* < 0.001), and the aHR was 3.66 for patients with ${\tilde{AHRR}}_{24hr}$ ≥ 20 bpm (95% CI: 2.05–6.52) when considering all demographic characteristics. 

## 4. Discussion

In this study, we presented the potential of using ${\tilde{AHRR}}_{24hr}$ acquired from the first Holter monitor to predict all-cause mortality among newly diagnosed AF patients. Higher ${\tilde{AHRR}}_{24hr}$ was associated with fewer all-cause mortality and cardiovascular mortality. In addition, the cut-off value of 20 bpm was found to be potentially useful in clinical practice to categorize AF patients into low and high risk groups. Additionally, we discovered that the combination of ${\tilde{AHRR}}_{24hr}$ and the CHA2DS2-VASc score had a higher association with the all-cause mortality than the use of ${\tilde{AHRR}}_{24hr}$ or the CHA2DS2-VASc score alone, which was similar across our study and previous studies [2,24,25,26]. Though few prior studies provided comparisons between the CHA2DS2-VASc score and measurements on cardiac rhythm. Our work provides a feasible way to combine clinical features and electrophysiology profiles for AF patient classification.

The value of 24-h AHRR, which has been found related to the autonomic tone in various cardiovascular diseases [20,28,29,30], was also associated with the prognosis of AF in this study. However, ${\tilde{AHRR}}_{24hr}$ seemed more indicative of the all-cause mortality than 24-h AHRR. Among all 30 parameters examined in this study, ${\tilde{AHRR}}_{24hr}$ was the only covariate retained in the backward selection of the Cox regression model, and the C-statistic was the best among all AHRR parameters (Supplementary Materials Files S6 and S8). The 24-h AHRR could represent a variability in heart rate, but it only considers the maximum and minimum values of AHRR during 24-h, which may only contain partial information about HR during the daytime and/or nighttime. Nonetheless, the importance of nighttime and daytime HR may be different in evaluating autonomic tone, and nighttime HR was reported to be more associated with cardiovascular outcomes [19]. ${\tilde{AHRR}}_{24hr}$, the median hourly AHRR during the whole day, ensures the diurnal change on HR would be considered as a single parameter. Moreover, for AF, the ventricular electric activities may be rather chaotic as the summation and cancellation of waves in the atrium, and previous spectral analysis on 24-h electrocardiogram revealed the long and short-term components may have different clinical implications [31]. Short-term components may reflect the immediate response to external stimulus, while long-term components may reflect the influence of 24-h circadian rhythm and physiological regulatory mechanisms on AF and sinus rhythm [32,33,34,35]. The ${\tilde{AHRR}}_{24hr}$ may also contain information about long-term components and therefore become a feasible way to assess the regulation and cardiac rhythm among AF patients. For this reason, ${\tilde{AHRR}}_{24hr}$ may also contain information about the vastly different heart rates in several short periods and reflect patients’ adaptation and responsiveness of the autonomic system. This may be the reason why ${\tilde{AHRR}}_{24hr}$ is more associated with all-cause mortality in AF than 24-h AHRR.

Additionally, the concept and calculation of ${\tilde{AHRR}}_{24hr}$ are simple and easier to understand when compared to HRV-related parameters. The prognostic value of some HRV-related parameters has been reported among AF patients [5,6,16,36]. However, most of them were acquired from resting ECG only, and these parameters were hard to interpret in clinical practice. For example, Yamada et al. reported reduced ventricular response irregularity assessed by entropy measures from 24-h ambulatory ECG recordings had higher mortality among outpatients with chronic AF [15]. Hämmerle et al. found that HRV indices from 5-min recordings, such as HRVI and RMSSD, could be a valuable predictor of cardiovascular mortality among AF patients [6]. In contrast, approximate entropy (ApEn), a nonlinear parameter, was more associated with prognosis in AF patients with CHF than the standard deviation of all normal RR intervals (SDNN) and RMSSD [16]. Though the idea of entropy may not be easily understood. We also examined the similar nonlinear analyses in this study: sample entropy and concordance entropy, in addition to SDNN and RMSSD. Although the correlations of these parameters between prognosis were similar to previous studies, these parameters were not statistically significant in a multivariate Cox regression and not selected in the final regression model (Supplementary Materials File S3). Conversely, the HRVI could also reflect the irregularity of RR intervals, and the feasibility in clinical practice seemed better than SDNN and RMSSD [6]. The association between ${\tilde{AHRR}}_{24hr}$ and irregularity of ventricular response may be speculated in our work as well as the work of Cubbon et al. [20]. ${\tilde{AHRR}}_{24hr}$ may be able to substitute HRV parameters for its relative simplicity and technical feasibility. In addition, our study shows the cut-off value of 20 bpm could be used as the threshold of ${\tilde{AHRR}}_{24hr}$ for mortality risk stratification among AF patients. The cut-off value of most HRV measurements has yet to be well-defined, and this problem may be caused by the relatively complicated calculations of HRV measurements, though many studies demonstrate the role of HRV in evaluating heart diseases [6,9,11,30,37,38].

Nevertheless, there are still some limitations of this study. First, this was a retrospective study aiming at the first Holter monitor of AF patients, and therefore a prospective study is required to avoid the potential bias towards patients selected to receive examinations. Regardless, the covariates with outcome impact and the implications from screening multiple parameters of the Holter monitor could be important references to further prospective studies. Secondly, the medication used after the Holter monitor examination was not considered in this study and may become an influential factor since this analysis was carried out by using the first Holter monitor of enrolled subjects. Finally, given that the subjects included in this study were all Taiwanese, the generalizability of using ${\tilde{AHRR}}_{24hr}$ among AF patients may be insufficient, especially when thromboembolic events are higher among subjects living in western countries as compared to eastern countries.

## 5. Conclusions

We demonstrated the association between ${\tilde{AHRR}}_{24hr}$ and all-cause mortality by screening multiple Holter monitor-derived parameters, and discovered that the cut-off value of 20 bpm could be considered a reference for mortality risk stratification among AF patients. However, prospective clinical studies are needed to validate the use of ${\tilde{AHRR}}_{24hr}$ to predict all-cause mortality among AF patients.

## Figures and Tables

**Table 1:** Baseline characteristics of enrolled atrial fibrillation patients with the first 24-h Holter cardiac rhythm monitor done during 1 January 2010–31 July 2014 (*n* = 521) (all cases were followed from the examination day to 30 June 2017).

Covariates	All Patients (*n* = 521)	Alive (*n* = 416)	Deceased (*n* = 105)	*p*
Age (years)	69.4 (13.1)	67.6 (12.9)	76.7 (11.4)	<0.001
<75	311 (59.7%)	277 (66.6%)	34 (32.4%)	<0.001
≥75	210 (40.3%)	139 (33.4%)	71 (67.6%)	
Gender				
Female	210 (40.3%)	158 (38.0%)	52 (49.5%)	0.031
Male	311 (59.7%)	258 (62.0%)	53 (50.5%)	
Co-morbidities				
Hypertension	298 (57.3%)	229 (55.2%)	69 (65.7%)	0.051
Diabetes mellitus	132 (25.4%)	98 (23.6%)	34 (32.4%)	0.065
Heart failure	154 (29.6%)	105 (25.3%)	49 (46.7%)	<0.001
Stroke	93 (17.9%)	64 (15.4%)	29 (27.6%)	0.004
Vascular diseases	69 (13.3%)	48 (11.6%)	21 (20.0%)	0.023
CHA_2_DS_2_-VASc score	3.1 (1.9)	2.8 (1.8)	4.2 (1.8)	<0.001
Medications				
Anticoagulant	326 (62.6%)	277 (66.6%)	49 (46.7%)	<0.001
Antiarrhythmic agents	142 (27.4%)	116 (28.0%)	26 (25.0%)	0.54
RAAS blockers	363 (69.8%)	295 (70.9%)	68 (65.4%)	0.27
Calcium channel blocker	276 (53.2%)	213 (51.3%)	63 (60.6%)	0.091
β-blocker	377 (72.5%)	314 (75.5%)	63 (60.6%)	0.002
Digoxin	222 (42.7%)	168 (40.4%)	54 (51.9%)	0.033
Physio-biochemical profiles				
MAP (mmHg)	93.6 (14.3)	93.4 (14.0)	94.3 (15.6)	0.59
Resting heart rate (bpm)	80.7 (20.4)	80.7 (20.2)	80.5 (21.3)	0.91
Cholesterol (mg/dL)	162.5 (35.3)	165.4 (34.9)	150.9 (34.7)	<0.001
Triglyceride (mg/dL)	105.7 (62.1)	106.3 (62.2)	103.1 (62.1)	0.65
Creatinine (mg/dL)	1.3 (1.3)	1.1 (0.9)	2.0 (2.3)	<0.001
eGFR (mL/min)	72.3 (37.2)	76.8 (35.4)	54.8 (39.0)	<0.001
LVEF (%)	58.7 (15.2)	58.7 (14.7)	58.6 (17.1)	0.94
Holter parameters				
${\tilde{AHRR}}_{24hr}$ (bpm)	22.8 (8.4)	24.0 (8.3)	18.4 (6.8)	<0.001
24-h AHRR (bpm)	58.1 (21.8)	60.2 (21.5)	49.6 (21.2)	<0.001

Abbreviations: ambulatory heart rate range (AHRR); estimated Glomerular filtration rate (eGFR); left ventricular ejection fraction (LVEF); mean arterial pressure (MAP); and renin-angiotensin-aldosterone system (RAAS).

**Table 2:** Univariate and multivariate Cox regression on different models.

Covariates	Univariate			Multivariate		
	Unadjusted HR (95% CI)		*p*	Adjusted HR (95% CI)		*p*
Age (years)						
<75	reference			reference		
≥75	3.87	(2.57–5.81)	<0.001	1.62	(0.51–5.12)	0.413
Gender						
Female	reference			reference		
Male	0.60	(0.41–0.88)	0.010	1.21	(0.47–3.14)	0.697
Co-morbidities						
Hypertension	1.54	(1.03–2.31)	0.036	0.88	(0.29–2.70)	0.821
Diabetes mellitus	1.49	(0.99–2.24)	0.054	0.98	(0.37–2.63)	0.974
Heart failure	2.23	(1.52–3.27)	<0.001	1.48	(0.58–3.76)	0.410
Stroke	1.78	(1.16–2.71)	0.008	0.89	(0.19–4.04)	0.876
Vascular disease	1.68	(1.04–2.71)	0.035	0.72	(0.26–2.01)	0.531
CHA_2_DS_2_-VASc score	1.43	(1.30–1.56)	<0.001	1.30	(0.59–2.84)	0.518
Medications						
Anticoagulant	0.45	(0.31–0.66)	<0.001	0.44	(0.28–0.69)	<0.001
Antiarrhythmic agents	0.91	(0.59–1.43)	0.695	0.96	(0.56–1.66)	0.892
RAAS blockers	0.79	(0.52–1.20)	0.270	0.68	(0.41–1.14)	0.141
Calcium channel blocker	1.39	(0.94–2.06)	0.095	0.77	(0.43–1.39)	0.390
β-blocker	0.57	(0.38–0.84)	0.005	0.57	(0.35–0.93)	0.024
Digoxin	1.47	(1.00–2.15)	0.051	2.07	(1.25–3.42)	0.005
Physio-biochemical profiles						
MAP (increase per 10 mmHg)	1.06	(0.92–1.22)	0.450	1.07	(0.91–1.26)	0.427
Resting heart rate (increase per 10 bpm)	0.99	(0.90–1.10)	0.903	1.02	(0.89–1.16)	0.804
Cholesterol (increase per10 mg/dL)	0.89	(0.83–0.94)	<0.001	0.92	(0.85–1.00)	0.064
Triglyceride (increase per 10 mg/dL)	0.99	(0.96–1.03)	0.660	1.01	(0.97–1.04)	0.727
Creatinine (mg/dL)	1.26	(1.18–1.34)	<0.001	1.12	(0.99–1.28)	0.073
eGFR (increase per 10 mL/min)	0.78	(0.71–0.87)	<0.001	0.93	(0.80–1.07)	0.296
LVEF (increase per 10%)	0.99	(0.86–1.14)	0.889	1.07	(0.90–1.26)	0.448
Holter parameters						
${\tilde{AHRR}}_{24hr}$ (Reduction per 10 bpm)	2.44	(1.82–3.23)	<0.001	2.70	(1.75–4.17)	<0.001

Abbreviations: ambulatory heart rate range (AHRR), estimated Glomerular filtration rate (eGFR), left ventricular ejection fraction (LVEF), mean arterial pressure (MAP), and renin-angiotensin-aldosterone system (RAAS).

**Table 3:** Baseline characteristics of AF patients stratified by ${\tilde{AHRR}}_{24hr}$ < 20 or ≥ 20 bpm.

Covariates	${\tilde{AHRR}}_{24hr}\geq 20$ (*n* = 305)	${\tilde{AHRR}}_{24hr}<20$ (*n* = 216)	*p*
Age (years)	68.1 (13.1)	71.2 (12.9)	0.009
<75	193 (63.3%)	118 (54.6%)	0.047
≥75	112 (36.7%)	98 (45.4%)	
Gender			
Female	110 (36.1%)	100 (46.3%)	0.019
Male	195 (63.9%)	116 (53.7%)	
Co-morbidities			
Hypertension	162 (53.3%)	136 (63.0%)	0.028
Diabetes mellitus	68 (22.4%)	64 (29.6%)	0.061
Heart failure	83 (27.3%)	71 (32.9%)	0.17
Stroke	50 (16.4%)	43 (19.9%)	0.31
Vascular disease	29 (9.5%)	40 (18.5%)	0.003
CHA_2_DS_2_-VASc score	2.8 (1.8)	3.5 (1.9)	<0.001
Medications			
Anticoagulant	192 (63.0%)	134 (62.0%)	0.83
Antiarrhythmic agents	80 (26.4%)	62 (28.8%)	0.54
ACEi/ARB	205 (67.4%)	158 (73.1%)	0.16
Calcium channel blocker	150 (49.5%)	126 (58.3%)	0.047
β-blocker	212 (69.7%)	165 (76.4%)	0.094
Digoxin	119 (39.1%)	103 (47.7%)	0.052
Physio-biochemical profiles			
MAP (mmHg)	94.4 (13.8)	92.4 (14.9)	0.12
Resting heart rate (bpm)	82.9 (20.6)	77.6 (19.8)	0.003
Cholesterol (mg/dL)	165.2 (34.2)	158.7 (36.5)	0.045
Triglyceride (mg/dL)	104.6 (57.5)	107.2 (68.2)	0.65
Creatinine (mg/dL)	1.0 (0.5)	1.6 (1.9)	<0.001
eGFR (mL/min)	76.4 (33.6)	66.6 (41.2)	0.003
LVEF (%)	58.1 (14.4)	59.6 (16.1)	0.26
Mortality			
All-cause mortality	34 (11.1%)	71 (32.9%)	<0.001
Cardiovascular mortality	20 (6.6%)	45 (20.8%)	<0.001

Abbreviations: angiotensin-converting enzyme inhibitor (ACEi), ambulatory heart rate range (AHRR), angiotensin receptor blocker (ARB), estimated Glomerular filtration rate (eGFR), left ventricular ejection fraction (LVEF), and mean arterial pressure (MAP).

## Data Availability

Data sharing is not applicable due to ethical regulations from the Institutional Review Board.

## Supplementary Materials

The following are available online at https://www.mdpi.com/article/10.3390/jpm11111202/s1, File S1: The detailed calculation process of parameters used in this study. File S2: Parameters of ambulatory heart rate (AHR) obtained from Holter ECG of all eligible atrial fibrillation patients. All continuous variables were presented as mean (standard deviation). File S3: (A) Univariate, (B) multivariate and (C) backward selection survival analysis on AHR parameters. File S4: (A) Univariate, (B) multivariate and (C) backward selection survival analysis on F-wave parameters. File S5: (A) Univariate, (B) multivariate and (C) backward selection survival analysis on RR parameters. File S6: (A) Multivariate and (B) backward stepwise variable selection survival analysis on all selected candidate parameters and demographic features. File S7: The correlation between ${\tilde{AHRR}}_{24hr}$ and 24-h AHRR. File S8: (A) Validation and (B) calibration of all candidate Holter parameters. File S9: Sensitivity test for associations between candidate covariates (demographic characteristics, ${\tilde{AHRR}}_{24hr}$) and two endpoints (all-cause mortality, cardiovascular mortality).
