# Supplementary material for: Is the Median Hourly Ambulatory Heart Rate Range Helpful in Stratifying Mortality Risk among Newly Diagnosed Atrial Fibrillation Patients?
Source: J Pers Med. 2021 Nov 14;11(11):1202. doi: 10.3390/jpm11111202 (PMC8622712; doi:10.3390/jpm11111202)
Supplement: Supplementary file 1 [file jpm-11-01202-s001.zip › jpm-1461501-supplementary.pdf]

Supplementary material S1. The detailed calculation process of parameters used in this study.

Ambulatory heart rate range

The ambulatory heart rate range (AHRR) index is calculated hourly as the difference between the maximum and minimum heart rate measurements made in that hour. The 24-hour AHRR index is obtained by taking the median of the 24-hour values. The AHRR index is intended to measure the physiological fitness of the heart.

Statistical indices

We view the 1440 measurements as having been sampled from distribution and study the properties of this distribution. The following statistical indices are considered. The standard deviation (SD) quantifies the variation in the data set by averaging the squared distance of each data point to the center (mean) of the distribution. The square-root function is applied. The population standard deviation is used.

The coefficient of variation (CV) describes the standard deviation of the distribution as a fraction of its mean. It is meant to account for the possibility that heart rate fluctuation may naturally increase with heart rate.

The signal-to-noise ratio (SNR) also accounts for the fact that a subject's heart rate variability may be a function of his or her resting heart rate. For example, it may be the case that individuals with a higher resting heart rate naturally have larger deviations in heart rate from minute to minute. The name comes from the field of image processing. We calculate the

value as the median divided by the modified standard deviation, where the center of the data is interpreted to be the median.

We also evaluate the sample skewness. A dataset is skewed (or non-symmetric) if its median and mean are different. If we view the data as having been sampled from a distribution, a skewness index should quantify how "tilted" this distribution is compared to a normal distribution. A positive value indicates that the distribution leans to the right, and a negative value indicates that the distribution leans to the left.

Recurrence indices

A recurrence plot is an embedding of the time series into high-dimensional space via the lag map. One may obtain the geometric properties of a recurrence plot using the singular value decomposition, principal component analysis, or the diffusion maps algorithm. These geometric properties describe the uniformity of the original time series[37].

A recurrence plot obtained from the lag map of order 2 is referred to as the Poincaré plot. Several indices derived from the Poincaré plot have been considered in the literature. In the end, these indices, which quantify the plot's geometric properties, describe the complexity of the original time series[38].

To calculate the indices AHRDay and AHRNight, we first need to cyclically shift the 24-hour ECG signal so that the first sample occurs just midnight. After doing so, we recalculate the time series H using the above definition (with the shifted peaks), and we set

$$\text{AHRDay} = \max_{11\times 60\leq n\leq 16\times 60} H(n) - \min_{11\times 60\leq n\leq 16\times 60} H(n)$$

$$\text{AHRNight} = \max_{2\times 60 \le n \le 3\times 60} H(n) - \min_{2\times 60 \le n \le 3\times 60} H(n)$$

That is, we estimate the AHR over the periods between 11:00 a.m. and 4:00 p.m. and 2:00 a.m. and 3:00 a.m. Additionally, Singular Value 1 refers to the largest singular value of the Poincaré plot, and Singular Value 2 refers to the second-largest singular value of the Poincaré plot.

Supplementary material S2. Parameters of ambulatory heart rate (AHR) obtained from Holter ECG of all eligible atrial fibrillation patients. All continuous variables were presented as mean (standard deviation).

Parameters	All patients (n=521)	Alive patients (n=416)	Deceased patients (n=105)	*P*
		AHR parameters		
$\widetilde{AHRR}_{24hr}$	22.833 (8.368)	23.961 (8.348)	18.365 (6.841)	<0.001
24-h AHRR	58.079 (21.814)	60.212 (21.464)	49.629 (21.225)	<0.001
AHRR (daytime)	69.879 (39.891)	71.380 (40.274)	63.933 (37.938)	0.087
AHRR (nighttime)	18.902 (10.410)	19.337 (10.383)	17.181 (10.387)	0.058
Coefficient of Variation (nighttime)	0.056 (0.030)	0.057 (0.030)	0.050 (0.030)	0.021
Concordance Entropy (nighttime)	0.614 (0.039)	0.616 (0.038)	0.604 (0.043)	0.006
Mean HR (nighttime)	74.757 (19.461)	73.382 (18.793)	80.188 (21.136)	0.001
Median HR (nighttime)	74.109 (19.474)	72.714 (18.784)	79.619 (21.205)	0.001
Sample Entropy (nighttime)	0.427 (0.151)	0.431 (0.152)	0.415 (0.146)	0.37
Singular Value1 (nighttime)	6.629 (4.769)	6.684 (4.751)	6.410 (4.858)	0.60
Singular Value2 (nighttime)	3.509 (1.850)	3.578 (1.826)	3.237 (1.927)	0.091
Standard Deviation of HR (nighttime)	4.081 (2.366)	4.142 (2.360)	3.841 (2.385)	0.25
Teager (nighttime)	8.975 (13.493)	9.150 (13.527)	8.284 (13.401)	0.56
		F-wave parameters		
Concordance Entropy (nighttime)	0.428 (0.016)	0.426 (0.015)	0.432 (0.016)	<0.001
Dominant Frequency (nighttime)	6.200 (1.175)	6.227 (1.159)	6.091 (1.236)	0.29
Amplitude (nighttime)	0.028 (0.016)	0.029 (0.016)	0.024 (0.012)	<0.001
Power (nighttime)	14.210 (15.663)	15.187 (16.436)	10.347 (11.401)	0.005
Sample Entropy (nighttime)	0.448 (0.040)	0.448 (0.039)	0.446 (0.047)	0.59
Singular Value1 (nighttime)	0.049 (0.029)	0.051 (0.030)	0.041 (0.022)	0.001
Singular Value2 (nighttime)	0.040 (0.022)	0.041 (0.023)	0.034 (0.018)	0.003
Skewness (nighttime)	-0.660 (2.580)	-0.700 (2.500)	-0.498 (2.881)	0.47
Standard Deviation (nighttime)	0.032 (0.016)	0.033 (0.016)	0.029 (0.014)	0.021
Teager Energy (nighttime)	0.072 (0.078)	0.078 (0.082)	0.049 (0.054)	<0.001
		RR parameters		
Coefficient of Variation (nighttime)	0.224 (0.064)	0.229 (0.063)	0.208 (0.065)	0.003
Concordance Entropy (nighttime)	0.601 (0.029)	0.602 (0.028)	0.596 (0.031)	0.046
Mean RR (nighttime)	0.857 (0.224)	0.870 (0.222)	0.803 (0.225)	0.006
Median RR (nighttime)	0.830 (0.227)	0.843 (0.224)	0.781 (0.232)	0.012
RMSSD (nighttime)	0.258 (0.103)	0.268 (0.104)	0.219 (0.092)	<0.001
SDNNI (nighttime)	14.360 (5.456)	14.402 (5.358)	14.196 (5.853)	0.73
Sample Entropy (nighttime)	0.213 (0.057)	0.215 (0.056)	0.204 (0.062)	0.067
Singular Value1 (nighttime)	37.928 (13.179)	38.054 (12.828)	37.431 (14.540)	0.67
Singular Value2 (nighttime)	17.147 (8.505)	16.986 (8.325)	17.783 (9.198)	0.39
SDNN (nighttime)	0.191 (0.072)	0.197 (0.072)	0.164 (0.063)	<0.001
Teager (nighttime)	27.789 (28.758)	26.799 (27.682)	31.702 (32.518)	0.12
Time Weighted Mean (nighttime)	76.488 (19.891)	75.148 (19.238)	81.786 (21.577)	0.002
Time Weighted Median (nighttime)	75.271 (19.946)	73.903 (19.206)	80.676 (21.913)	0.002
pRR50 (nighttime)	0.373 (0.077)	0.377 (0.077)	0.356 (0.077)	0.011

Abbreviations: RMSSD, root mean of successive differences between N-N intervals; SDNN, standard deviation of all N-N intervals; SDNNI, SDNN index (averaged 5-min SDNN)

Supplementary material S3. (A) Univariate, (B) multivariate and (C) backward selection survival analysis on AHR parameters.

Parameters	(A) HR (95% CI)		*P*	(B) HR (95% CI)		*P*	(C) HR (95% CI)		*P*
$\widetilde{AHRR}_{24hr}$	0.01	(0.00-0.06)	<0.001	0.00	(0.00–0.01)	0.000	0.00	(0.00–0.01)	0.000
24-h AHRR	0.05	(0.01-0.18)	<0.001	8.47	(0.58–124.67)	0.119			
AHRR (daytime)	0.36	(0.14-0.93)	0.035	1.00	(0.38–2.61)	0.999			
AHRR (nighttime)	0.29	(0.09-0.96)	0.043	0.03	(0.00–1.70)	0.089			
Coefficient of Variation (nighttime)	0.19	(0.05-0.68)	0.011	257.19	(6.91–9,578.32)	0.003	174.09	(5.15–5,885.29)	0.004
Singular Value1 (nighttime)	0.74	(0.28-1.98)	0.554						
Singular Value2 (nighttime)	0.33	(0.10-1.08)	0.067						
Concordance Entropy (nighttime)	0.22	(0.08-0.62)	0.004	0.27	(0.07–1.00)	0.051			
Sample Entropy (nighttime)	0.65	(0.23-1.86)	0.419						
Teager (nighttime)	0.72	(0.27-1.93)	0.514						
Mean HR (nighttime)	7.17	(2.31-22.26)	0.001	0.00	(0.00–1,806.17)	0.299			
Median HR (nighttime)	7.27	(2.36-22.36)	0.001	640,134.38	(0.07–5.94e+12)	0.102	138.67	(20.00–961.41)	0.000
Standard Deviation of HR (nighttime)	0.49	(0.16-1.47)	0.205						

Supplementary material S4. (A) Univariate, (B) multivariate and (C) backward selection survival analysis on F-wave parameters.

Parameters	(A) HR (95% CI)		*P*	(B) HR (95% CI)		*P*	(C) HR (95% CI)		*P*
Dominant Frequency (nighttime)	0.46	(0.12–1.74)	0.251						
Power (nighttime)	0.26	(0.11–0.61)	0.002	6.86	(0.80–58.95)	0.079			
Sample Entropy (nighttime)	0.79	(0.22–2.80)	0.709						
Amplitude (nighttime)	0.16	(0.06–0.43)	<0.001	0.16	(0.01–3.35)	0.240			
Skewness (nighttime)	1.52	(0.89–2.60)	0.129						
Teager Energy (nighttime)	0.20	(0.09–0.47)	<0.001	2.97	(0.20–44.11)	0.429			
Standard Deviation (nighttime)	0.26	(0.09–0.75)	0.013	74,339.89	(70.76–78096799.54)	0.002	129,170.49	(312.08–53464591.43)	0.000
Singular Value1 (nighttime)	0.16	(0.06–0.44)	<0.001	0.02	(0.00–3.62)	0.134	0.01	(0.00–0.53)	0.025
Singular Value2 (nighttime)	0.17	(0.06–0.50)	0.001	0.00	(0.00–0.14)	0.014	0.00	(0.00–0.05)	0.006
Concordance Entropy (nighttime)	13.91	(2.91–66.51)	0.001	3.19	(0.18–55.74)	0.426			

Supplementary material S5. (A) Univariate, (B) multivariate and (C) backward selection survival analysis on RR parameters.

	(A)			(B)			(C)		
Parameters	HR (95% CI)		*P*	HR (95% CI)		*P*	HR (95% CI)		*P*
SDNNI (nighttime)	0.88	(0.26–2.94)	0.832						
pRR50 (nighttime)	0.32	(0.17–0.61)	0.001	2.45	(0.39–15.58)	0.342			
RMSSD (nighttime)	0.07	(0.02–0.22)	<0.001	0.07	(0.00–23.20)	0.373			
Coefficient of Variation (nighttime)	0.13	(0.04–0.49)	0.002	0.01	(0.00–68.69)	0.309	0.09	(0.02–0.34)	0.001
Singular Value1 (nighttime)	0.80	(0.21–3.04)	0.749						
Singular Value2 (nighttime)	1.56	(0.46–5.30)	0.477						
Concordance Entropy (nighttime)	0.26	(0.08–0.84)	0.025	1.23	(0.12–12.62)	0.862			
Sample Entropy (nighttime)	0.53	(0.29–0.97)	0.041	0.64	(0.17–2.38)	0.502			
Teager (nighttime)	1.78	(0.80–3.96)	0.159						
Mean RR (nighttime)	0.13	(0.03–0.50)	0.003	134.70	(0.00–41948958.64)	0.447			
Time Weighted Mean (nighttime)	6.64	(2.12–20.76)	0.001	1,084.80	(0.00–1.87e+10)	0.411			
Median RR (nighttime)	0.17	(0.05–0.60)	0.006	0.01	(0.00–485.55)	0.414			
Time Weighted Median (nighttime)	6.70	(2.15–20.94)	0.001	0.03	(0.00–270,442.81)	0.667	8.53	(2.82–25.78)	0.000
SDNN (nighttime)	0.08	(0.03–0.24)	<0.001	27.18	(0.00–250,229.98)	0.478			

Supplementary material S6. (A) Multivariate and (B) backward stepwise variable selection survival analysis on all selected candidate parameters and demographic features.

Parameters	(A) Adjusted HR (95% CI)		*P*	(B) Adjusted HR (95% CI)		*P*
AHR						
$\widetilde{AHRR}_{24hr}$	0.00	(0.000–0.021)	0.000	0.00	(0.000–0.017)	0.000
Coefficient of Variation (nighttime)	0.98	(0.192–5.008)	0.981			
Median AHR (nighttime)	1.31e+11	(5,662.247–3.012e+18)	0.003	9.36e+10	(4,522.875–1.938e+18)	0.003
FWAVE						
Standard Deviation (nighttime)	133.80	(0.068–262,661.640)	0.206			
Singular Value1 (nighttime)	0.03	(0.000–3.596)	0.151	0.01	(0.000–0.853)	0.042
Singular Value2 (nighttime)	0.10	(0.000–1,118.602)	0.628			
RR						
Coefficient of Variation (nighttime)	4.25	(0.581–31.068)	0.154			
Time Weighted Median (nighttime)	0.00	(0.000–0.005)	0.011	0.00	(0.000–0.007)	0.011

Supplementary material S7. The correlation between $\widetilde{AHRR}_{24hr}$ and 24-h AHRR.

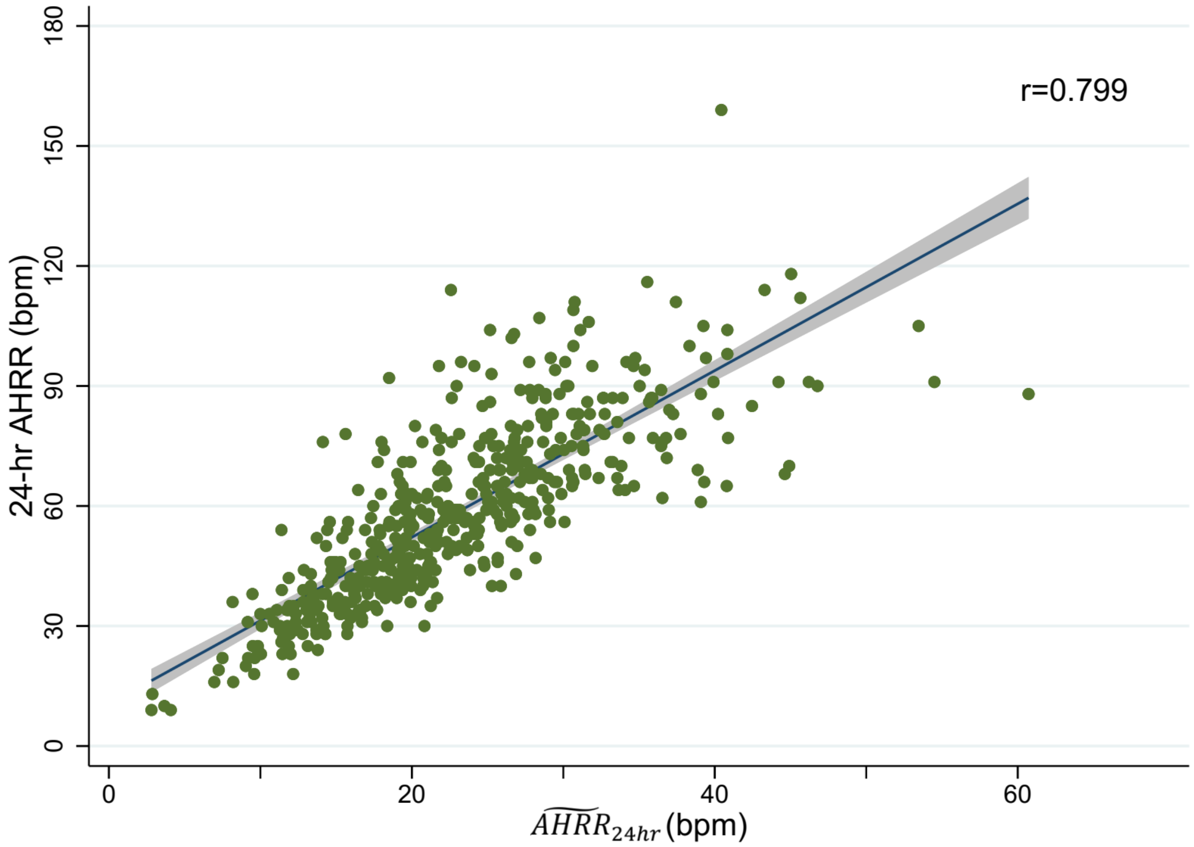

Supplementary material S8. (A) Validation and (B) calibration of all candidate Holter parameters.

	(A)Validation			(B) Calibration	
Parameters	C-statistic	95%CI	*P*	GoF	*P*
$\widetilde{AHRR}_{24hr}$	0.707	(0.658-0.756)	<0.001	1.899	0.168
24-h AHRR	0.656	(0.600-0.713)	<0.001	1.203	0.273
AHRR (daytime)	0.585	(0.527-0.643)	0.005	2.389	0.122
Median AHR (nighttime)	0.591	(0.533-0.649)	0.002	0.418	0.518
F-wave Singular Value1 (nighttime)	0.578	(0.524-0.631)	0.004	7.317	0.007
RR Time Weighted Median (nighttime)	0.585	(0.527-0.643)	0.005	0.163	0.686

Abbreviations: AHRR: ambulatory heart rate range; AUROC, the area under receiver operating curve; CI, confidence interval; GoF: goodness-of-fit

Supplementary material S9. Sensitivity test for associations between candidate covariates (demographic characteristics, $\widetilde{AHRR}_{24hr}$) and two endpoints (all-cause mortality, cardiovascular mortality)

	C-statistics	95% CI
Cardiovascular mortality		
All baseline	0.857	(0.801–0.912)
Sig. Baseline*	0.841	(0.781–0.900)
$\widetilde{AHRR}_{24hr}$	0.730	(0.669–0.791)
Baseline + $\widetilde{AHRR}_{24hr}$	0.887	(0.847–0.928)
1:1 subsampling (all-cause mortality)		
All baseline	0.633	(0.566–0.699)
Sig. Baseline	0.598	(0.536–0.660)
$\widetilde{AHRR}_{24hr}$	0.533	(0.474–0.592)
Baseline + $\widetilde{AHRR}_{24hr}$	0.671	(0.610–0.731)
5-fold cross-validation with 100 repeats (all-cause mortality)		
All baseline	0.665	(0.662–0.668)
Sig. Baseline	0.767	(0.765–0.769)
$\widetilde{AHRR}_{24hr}$	0.707	(0.706–0.708)
Baseline + $\widetilde{AHRR}_{24hr}$	0.694	(0.691–0.697)

Abbreviations: ambulatory heart rate range (AHRR); the area under receiver operating curve (AUROC); confidence interval (CI)

*Significant baseline parameters: Cholesterol, Digoxin, β-blockers, eGFR, anticoagulant and CHA_2DS_2-VASc
